# Supplementary material for: A GPAT1 Mutation in Arabidopsis Enhances Plant Height but Impairs Seed Oil Biosynthesis
Source: Int J Mol Sci. 2021 Jan 14;22(2):785. doi: 10.3390/ijms22020785 (PMC7829857; doi:10.3390/ijms22020785)
Supplement: Supplementary file 1 [file ijms-22-00785-s001.zip › Supplemental Figures.docx]

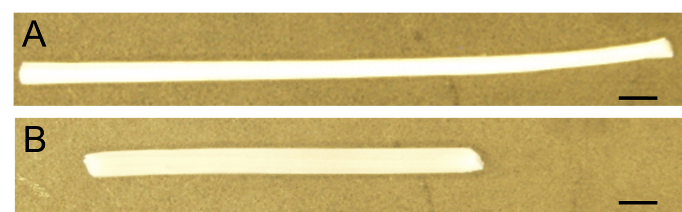


**Figure S1.** Analysis of GPAT1 expression in the middle and lower stems of Arabidopsis wild-type plants by GUS staining. A and B, expression analysis of GPAT1 by *Pro_GPAT1_-GUS*. No GUS activity was detected in the middle or lower stems. Bar = 1 mm.


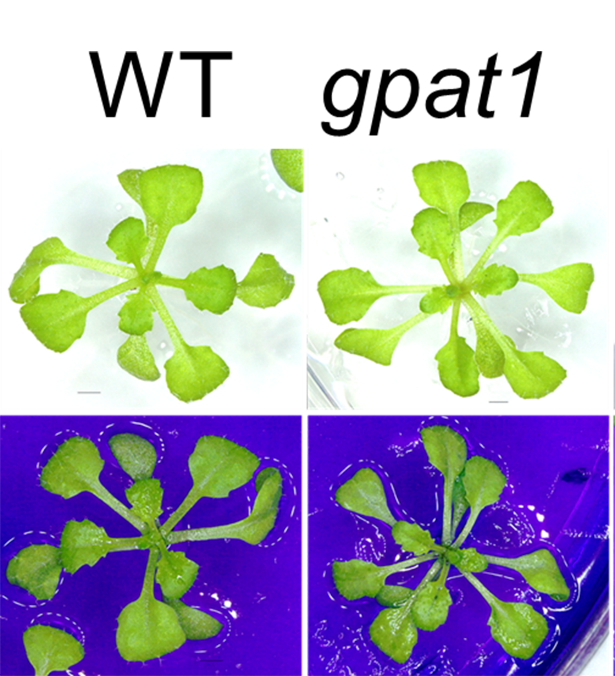


**Figure S2.** Permeability of cuticles of WT and *gpat1* mutant to toluidine blue. Seedlings were growed in MS medium for 15 days and then immersed for 2 min in 0.05% toluidine blue-O and rinsed with water. WT, wild type; Bar = 1 mm.


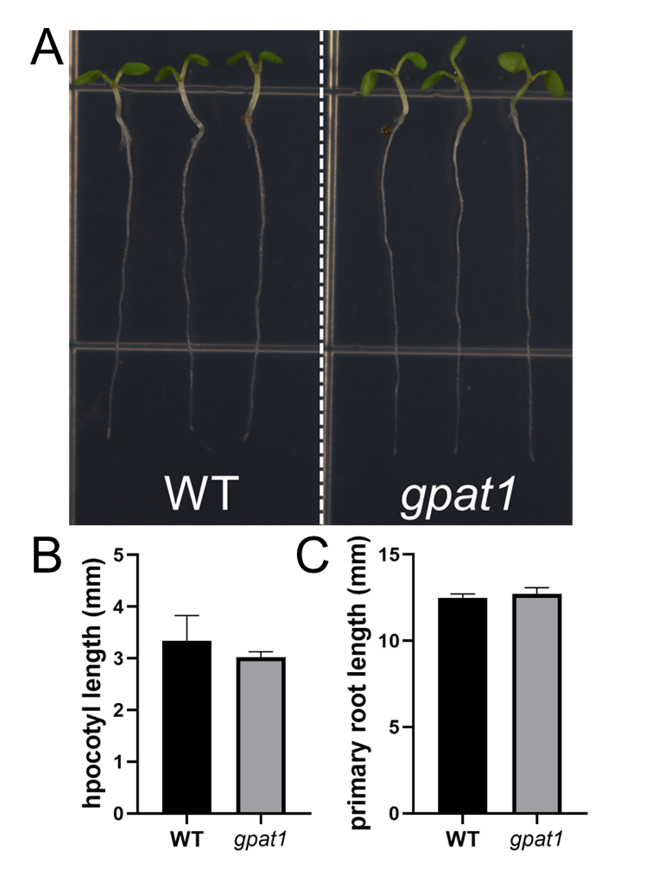


**Figure S3.** The effects of disruption of *GPAT1* gene on root development. A. 7-day-old seedlings of WT and *gpat1* mutant plants. B. Statistical data of hypocotyl length of WT and *gpat1* mutant plants. At least 25 seedlings were measured for each genotype. C. Statistical data of primary root length of WT and *gpat1* mutant plants. At least 25 seedlings were measured for each genotype. Values are means ± SE based on Student’s *t*-test.
