# Supplementary material for: A GPAT1 Mutation in Arabidopsis Enhances Plant Height but Impairs Seed Oil Biosynthesis
Source: Int J Mol Sci. 2021 Jan 14;22(2):785. doi: 10.3390/ijms22020785 (PMC7829857; doi:10.3390/ijms22020785)
Supplement: Supplementary file 1 [file ijms-22-00785-s001.zip › Supplemental Table S1.docx]

**Table S1** Details of gene primers used in this article.

| Name |  | Sequence (5’-3’) | Description |
| --- | --- | --- | --- |
| *GPAT1pro* | Forward | 5’-CTGCAGGCATGCAAGCTTAATTATCATCGAATTTTC-3’ | pcambia1301-*GPAT1pro-GUS*  (*pGPAT1pro-GUS*) |
|  | Reverse | 5’-CCCTCAGATCTACCATGGAGCTATGGCGTAGAGAGA-3’ |  |
| *GPAT1 sgRNA* | Forward | 5’-GATTGGAAACCAGAACCAGAAGCT-3’ | pAtU6-26:sgRNA-23p35S:Cas9 pBlunt |
|  | Reverse | 5’-AAACAGCTTCTGGTTCTGGTTTCC-3’ |  |
| *GPAT1 genomic* | Forward | 5’-GGATCCTCTAGAGTCGACCTGCAGGTGTCTAAATATTGAAAGG-3’ | pcambia1300-*GPAT1 genomic* |
|  | Reverse | 5’-AGTGCCAAGCTTGCATGCCTGCAGATGGACTATACAAATCCAG-3’ |  |
| *SALK_052352* | LP | 5’-TCGTGTGAGGGAAAGGTAATG-3’ | Mutant identification |
|  | RP | 5’- AGCCTAAGCAAGTTCTCCGAG-3’ |  |
| *GPAT1* | Forward | 5’-CCTCCTCGACCCAGTTTTCC-3’ | *gpat1*-RT-PCR |
|  | Reverse | 5’-TGGCACTAAAGCGACAGGTT-3’ |  |
| *GPAT1* | Forward | 5’-TTCTGGTTCTGGTTTCGTCCTGAAAC-3’ | *gpat1-c1*-RT-PCR |
|  | Reverse | 5’-CTTATCCCTCCTTGTGAGATTAGTG-3’ |  |
| *LBb1.3* | 5’-ATTTTGCCGATTTCGGAAC-3’ | | Mutant identification |
| *gpat1-c1* | Forward | 5’-CCACACGTTCTTCCCTTACTTC -3’ | CRISPR identification |
|  | Reverse | 5’-CTCTAGGCAATGCGTTGTTC-3’ |  |
| *GGPPS2* | Forward | 5’-AATGATCCACACAATGTCGTTG-3’ | real-time qRT-PCR |
|  | Reverse | 5’-TTCACCGAAAACTTTGTGGTTC-3’ |  |
| *GGPPS4* | Forward | 5’-GAATTGGCTAGGTCTATTGGGA-3’ | real-time qRT-PCR |
|  | Reverse | 5’-AACTCCAAATGCTCTAATCCGA-3’ |  |
| *GA3ox1* | Forward | 5’-GCCTGCTATGTTAACAGATGTG-3’ | real-time qRT-PCR |
|  | Reverse | 5’-GAAGAGGAGATCGTCTTTAGGG-3’ |  |
| *GA20ox3* | Forward | 5’-CTACTAGACTCGTCTCAAAGGC-3’ | real-time qRT-PCR |
|  | Reverse | 5’-AAGAGTCCATATGCAGATAGGC-3’ |  |
| *GA20ox5* | Forward | 5’-AGACTGTCTGCCCCTATTTAAC-3’ | real-time qRT-PCR |
|  | Reverse | 5’-ACCACTAGAAAAGTACCGTGAG-3’ |  |
| *GA2ox1* | Forward | 5’-TCTAACAACACTTCTGGTCTCC-3’ | real-time qRT-PCR |
|  | Reverse | 5’-TTCTCTGAGTCAATGAAGGTCC-3’ |  |
| *GA2ox2* | Forward | 5’-CTGTGTGAAAGATGGAAGTTGG-3’ | real-time qRT-PCR |
|  | Reverse | 5’-GGTCCGCCGAAATATATCATTG-3’ |  |
| *GA2ox4* | Forward | 5’-TAAACTTCTTTGCTAAACCGGC-3’ | real-time qRT-PCR |
|  | Reverse | 5’-GAAACAGCAAATACTCGACCTC-3’ |  |
| *NPF3.1* | Forward | 5’-GAAACAGCAAATACTCGACCTC-3’ | real-time qRT-PCR |
|  | Reverse | 5’-GAAACAGCAAATACTCGACCTC-3’ |  |
| *GID1B* | Forward | 5’-AGAAGACCCTTGATGGCAAATA-3’ | real-time qRT-PCR |
|  | Reverse | 5’-CAAAGGGATTACATGCTGGATG-3’ |  |
| *XTH31* | Forward | 5’-ACAAAAACTTTAAGCTAGCGGG-3’ | real-time qRT-PCR |
|  | Reverse | 5’-GTTTGGGTATGGTCTCTTTTCG-3’ |  |
| *PME16* | Forward | 5’-GCTGGACTACTTACAAAACTGC-3’ | real-time qRT-PCR |
|  | Reverse | 5’-TGGAGTGTGTGTAGAGTGAATC-3’ |  |
| *PMEI6* | Forward | 5’-TAGCAACATCAACACAAACGAC-3’ | real-time qRT-PCR |
|  | Reverse | 5’-GTTTGGTGTCGGTTATGGTAAC-3’ |  |
|  |  |  |  |
| *RGP3* | Forward | 5’-ACTCCGCATTTCTTTAACACAC -3’ | real-time qRT-PCR |
|  | Reverse | 5’-GTCATGACTGCATCAACATACC -3’ |  |
| *XYN4* | Forward | 5’-TACTCTTTGGCCTCTAAGTTGG -3’ | real-time qRT-PCR |
|  | Reverse | 5’-CTCTTGCATCTTCTTTACCACG -3’ |  |
| *CSLB3* | Forward | 5’-TATGTTTGTGACTACTGCGGAT -3’ | real-time qRT-PCR |
|  | Reverse | 5’-CGCTGGATAATTCACTGCTAAC -3’ |  |
| *EXPA15* | Forward | 5’-CTTAAACGGTCAAGCATTGTCA -3’ | real-time qRT-PCR |
|  | Reverse | 5’-TAGCTGGAGCAATGTTGTTAGA -3’ |  |
| *EXT4* | Forward | 5’-TACTCTCCTCCTCCTGTTTACA -3’ | real-time qRT-PCR |
|  | Reverse | 5’-GGAGGAGGGGAGTAGTACTTAA -3’ |  |
| *RUBY* | Forward | 5’-GGATCATACAACCTCGCTAGAA -3’ | real-time qRT-PCR |
|  | Reverse | 5’-TTAGCAAAACATCTCCGTTTGG -3’ |  |
| *PER36* | Forward | 5’-TTGGTTCCATGGAAAACATTCC-3’ | real-time qRT-PCR |
|  | Reverse | 5’-TGAGTTTCCTATCGTGTGACTC -3’ |  |
| *ACTIN 2* | Forward | 5’-GACCTTGCTGGACGTGACCTTAC-3’ | real-time qRT-PCR |
|  | Reverse | 5’-GTAGTCAACAGCAACAAAGGAGAGC-3’ |  |
| *GPAT1* | Forward | 5’-CCCTCCTCGACCCAGTTTTC-3’ | real-time qRT-PCR |
|  | Reverse | 5’-AGTCTCTGCATTGCCTCACC-3’ |  |
|  |  |  |  |
