# Supplementary material for: A GPAT1 Mutation in Arabidopsis Enhances Plant Height but Impairs Seed Oil Biosynthesis
Source: Int J Mol Sci. 2021 Jan 14;22(2):785. doi: 10.3390/ijms22020785 (PMC7829857; doi:10.3390/ijms22020785)
Supplement: Supplementary file 1 [file ijms-22-00785-s001.zip › Supplemental Table S2.docx]

**Table S2**. Expression patterns of genes in selected pathways.

**Functional Category Gene ID Gene Name ^a^Fold Change *p*-adjust**

AT4G15560 DXS -0.6424 3.29 x 10^-38^

AT5G62790 DXR -0.2291 2.87 x 10^-4^

AT2G02500 MCT -0.0024 0.9926

AT2G26930 CMK 0.0652 0.7308

AT1G63970 MDS -0.2323 0.0110

AT5G60600 HDS -0.2167 1.91 x 10^-4^

AT4G34350 HDR -0.7023 6.81 x 10^-37^

AT3G02780 IPPI1 0.0539 0.6354

AT5G16440 IPPI2 -0.091 0.4948

AT5G47770 FPPS1 0.0098 0.9538

**MEP pathway** AT4G17190 FPPS2 -0.0152 0.9329

AT1G49530 GGPPS1 0.3017 0.9308

AT2G18620 GGPPS2 1.2563 2.06 x 10^-3^

AT2G18640 GGPPS3 4.7530 6.20 x 10^-2^

AT2G23800 GGPPS4 1.5513 2.43 x 10^-3^

AT3G14510 GGPPS5 0.6011 0.7553

AT3G14530 GGPPS6 0.6752 1.63 x 10^-7^

AT3G14550 GGPPS7 0.5003 2.01 x 10^-6^

AT3G20160 GGPPS8 0 1

AT3G29430 GGPPS9 -0.8946 1

AT3G32040 GGPPS10 2.9452 1

AT4G36810 GGPPS11 -1.1326 0.8335

AT4G38460 GGPPS12 0.4140 5.02 x 10^-9^

AT4G02780 CPS/GA1 -0.9282 0.1061

AT1G79460 KS/GA2 -0.3126 2.64 x 10^-5^

AT5G25900 KO/GA3 -0.0219 0.9018

AT1G05160 KAO1 0.2476 0.0296

AT2G32440 KAO2 -0.0616 0.6958

AT1G15550 GA3ox1/GA4 1.3190 3.65 x 10^-5^

AT1G80340 GA3ox2 0.3344 0.9142

**GA metabolism** AT4G21690 GA3ox3 0.7165 0.4022

AT1G80330 GA3ox4 -0.5386 0.1616

AT4G25420 GA20ox1 0.3885 1.03 x 10^-8^

AT5G51810 GA20ox2 -0.5449 2.98 x 10^-4^

AT5G07200 GA20ox3 1.8466 2.08 x 10^-11^

AT1G60980 GA20ox4 0.0671 1

AT1G44090 GA20ox5 -1.2931 1.85 x 10^-6^

AT1G78440 GA2ox1 -4.1461 9.42 x 10^-76^

AT1G30040 GA2ox2 1.0711 5.27 x 10^-21^

AT2G34555 GA2ox3 -0.8683 0.2453

AT1G47990 GA2ox4 -1.1554 0.0413

**Table S2**. *Cont.*

**Functional Category Gene ID Gene Name ^a^Fold Change *p*-adjus**

AT3G17203 GA2ox5 No No

AT1G02400 GA2ox6 -0.3365 0.6370

AT1G50960 GA2ox7 2.8442 0.2467

**GA metabolism** AT4G21200 GA2ox8 0.0389 0.9935

AT4G26420 GAMT1 -0.2081 0.0172

AT5G56300 GAMT2 -0.4497 9.44 x 10^-7^

AT5G24910 CYP714A1 -0.4705 3.02 x 10^-7^

AT5G24900 CYP714A2 0.5051 0.0669

AT1G68570 NPF3.1 1.8622 1.13 x 10^-131^

AT3G47960 NPF2.10 0.6771 2.92 x 10^-17^

**GA transport** AT5G50800 SWEET13 -0.3327 0.4566

AT4G25010 SWEET14 0.4010 0.7736

AT1G14920 GAI 0.1272 0.4748

AT3G05120 GID1A 0.1355 0.2596

**GA signaling** AT3G63010 GID1B 1.7400 2.10 x 10^-5^

AT5G27320 GID1C -0.3962 0.0366

AT4G24210 SLY1 0.0399 0.8794

AT5G48170 SLY2/SNE -0.0327 0.9874

AT3G25050 XTH3 1.8963 5.09 x 10^-4^

AT5G57530 XTH12 3.7167 0.0480

AT4G14130 XTH15 1.6513 1.93 x 10^-8^

AT4G30280 XTH18 1.8079 4.62 x 10^-4^

AT4G30290 XTH19 1.2030 2.26 x 10^-4^

AT4G25810 XTH23 1.4676 1.51 x 10^-6^

AT3G44990 XTH31 1.1909 1.69 x 10^-5^

AT1G10550 XTH33 1.2876 6.44 x 10^-5^

**cell wall organization** AT1G23200 PME6 1.5739 2.22 x 10^-6^

**or biogenesis**  AT1G02810 PME7 1.0591 2.44 x 10^-3^

**(up-regulated DEGs)**  AT2G36710 PME15 1.5360 8.60 x 10^-65^

AT2G43050 PME16 2.2244 4.25 x 10^-7^

AT5G18990 PME55 4.1205 9.80 x 10^-3^

AT5G51500 PME60 3.4539 5.65 x 10^-3^

AT2G47670 PMEI6 1.6810 5.65 x 10^-19^

AT1G09550 PAE1 1.8961 1.05 x 10^-4^

AT3G08900 RGP3 2.1478 5.60 x 10^-10^

AT5G50750 RGP4 1.3445 2.13 x 10^-8^

AT2G14690 XYN4 2.5353 5.03 x 10^-5^

AT2G32610 CSLB1 5.8304 8.07 x 10^-4^

AT2G32530 CSLB3 1.6485 1.30 x 10^-79^

AT2G37640 EXPA3 1.1299 4.60 x 10^-20^

AT2G03090 EXPA15 1.3783 2.84 x 10^-23^

AT1G76930 EXT4 1.5450 1.42 x 10^-3^

**Table S2**. *Cont.*

**Functional Category Gene ID Gene Name ^a^Fold Change *p*-adjus**

AT1G19900 RUBY 2.3440 4.97 x 10^-23^

AT2G41480 PER25 1.2009 0.0300

AT3G50990 PER36 1.7295 8.75 x 10^-3^

AT4G31500 CYP83B1 1.0827 6.84 x 10^-4^

AT3G57510 ADPG1 1.3150 4.05 x 10^-21^

**cell wall organization** AT2G41850 ADPG2 4.8307 0.03498

**or biogenesis** AT3G07970 QRT2 2.5493 0.0484

**(up-regulated DEGs)** AT5G01100 FRB1 1.8164 6.51 x 10^-8^

AT3G12500 CHI-B 1.7468 2.14 x 10^-15^

AT1G02720 GATL5 1.1920 0.0177

AT5G65590 DOF5.7 1.1567 8.47 x 10^-7^

AT2G15440 AT2G15440 1.0830 7.71 x 10^-10^

AT4G33820 AT4G33820 1.8889 1.59 x 10^-7^

AT2G47030 PME4 -2.5582 1.55 x 10^-30^

AT2G47040 PME5 -2.5687 1.59 x 10^-49^

AT2G26450 PME13 -2.0044 1.46 x 10^-8^

AT3G05610 PME21 -2.5889 9.61 x 10^-26^

AT5G27870 PME28 -2.8674 7.22 x 10^-5^

AT4G02300 PME39 -1.5888 6.36 x 10^-10^

AT5G07410 PME48 -2.5862 3.29 x 10^-8^

AT5G07430 PME50 -2.4325 2.61 x 10^-25^

AT3G17060 PME67 -2.3618 5.12 x 10^-5^

AT5G46960 PMEI12 -1.1143 1.46 x 10^-3^

AT1G69940 PPME1 -2.4632 0.01044

**cell wall organization** AT5G39260 EXPA21 -1.0089 8.19 x 10^-3^

**or biogenesis** AT5G39280 EXPA23 -1.1021 9.05 x 10^-4^

**(down-regulated DEGs)** AT5G39300 EXPA25 -1.0565 2.63 x 10^-4^

AT2G45110 EXPB4 -1.1631 5.13 x 10^-15^

AT2G33100 CSLD1 -1.3883 3.23 x 10^-3^

AT1G32180 CSLD6 -1.0910 0.0477

AT3G20580 COBL10 -1.9091 1.79 x 10^-6^

AT2G37720 TBL15 -1.2783 4.27 x 10^-11^

AT3G11550 CASP2 -1.4912 3.83 x 10^-3^

AT5G46330 FLS2 -1.0672 2.30 x 10^-19^

AT3G62170 VGDH2 -2.4478 1.41 x 10^-17^

AT1G02640 BXL2 -1.1372 7.25 x 10^-10^

AT1G14420 AT59 -2.1400 1.48 x 10^-12^

AT1G05250 PER2 -4.8053 0.0353

AT5G61680 AT5G61680 -4.4850 2.18 x 10^-3^

^a^Fold change: fold change between *gpat1* and wild type. Values mean log_2_ fold ratio. The data source comes from TAIR (http://www.arabidopsis.org/).
